# Tunable Interlayer Delocalization of Excitons in Layered Organic–Inorganic Halide Perovskites

**DOI:** 10.1021/acs.jpclett.3c02339

**Published:** 2023-11-20

**Authors:** Yinan Chen, Marina R. Filip

**Affiliations:** Department of Physics, University of Oxford, Clarendon Laboratory, Oxford OX1 3PU, U.K.

## Abstract

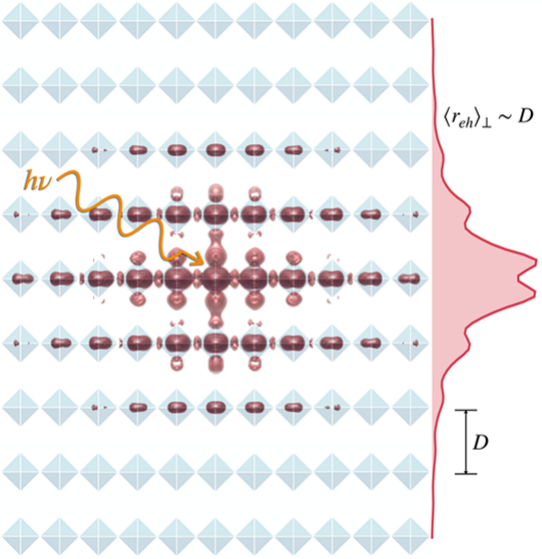

Layered organic–inorganic halide perovskites exhibit remarkable structural and chemical diversity and hold great promise for optoelectronic devices. In these materials, excitons are thought to be strongly confined within the inorganic metal halide layers with interlayer coupling generally suppressed by the organic cations. Here, we present an in-depth study of the energy and spatial distribution of the lowest-energy excitons in layered organic–inorganic halide perovskites from first-principles many-body perturbation theory, within the *GW* approximation and the Bethe–Salpeter equation. We find that the quasiparticle band structures, linear absorption spectra, and exciton binding energies depend strongly on the distance and the alignment of adjacent metal halide perovskite layers. Furthermore, we show that exciton delocalization can be modulated by tuning the interlayer distance and alignment, both parameters determined by the chemical composition and size of the organic cations. Our calculations establish the general intuition needed to engineer excitonic properties in novel halide perovskite nanostructures.

Layered organic–inorganic halide perovskites make up a structurally and chemically heterogeneous family^1^ of functional semiconductors with promising optoelectronic properties^2−4^ for devices, including solar cells,^2,5−7^ light-emitting diodes,^1,8,9^ photodetectors,^10^ photocatalysts,^11^ and lasers.^12^ Layered perovskites consist of an alternate stacking of inorganic layers of corner-sharing metal halide octahedra and large organic molecular cations,^9^ thereby displaying an exceptionally diverse range of structural configurations and chemical compositions. While they are formally bulk three-dimensional (3D) materials, the alternation of inorganic and organic layers is thought to lead to a quasi-two-dimensional behavior of electronic and optical properties.^3,13^ Interlayer electronic coupling and charge transfer are suppressed,^14−16^ and photoexcited electron–hole pairs (excitons) are strongly bound and localized within the inorganic layer, as a consequence of quantum confinement.^17−19^ Signatures of electron–phonon and exciton–phonon coupling are observed in photoluminescence spectra of layered perovskites that exhibit regularly spaced peaks.^20,21^ These strong interactions also likely give rise to the formation of self-trapped excitons, which have been attributed to broad photoluminescence spectra and white light emission in layered halide perovskites.^1^ Furthermore, recent experimental measurements report possible evidence of interlayer exciton transport,^22^ interlayer exciton delocalization,^23^ or charge transfer excitons^24^ in bulk layered perovskite systems, which may be explored in devices.^22−26^ These studies highlight the need for a systematic understanding of how the localization of excitons in layered organic–inorganic halide perovskites can be tuned through the exploration of the broad chemical and structural heterogeneity of this family of materials.

Correlation of photoexcited electron–hole pairs which are localized in spatially separated layers can be facilitated by the coupling of single-particle wave functions from neighboring layers.^27^ It is also typically associated with a type II energy band alignment in a heterostructure^27−29^ and/or with spin-valley locking occurring as a result of broken inversion symmetry.^30^ For example, low-dimensional van der Waals bound systems, including those based on transition metal dichalcogenides (TMDCs), are known to facilitate formation of long-lived excitons that delocalize across different layers, such as dipolar and quadrupolar interlayer excitons reported in hetero bi- and trilayers of TMDCs;^28,31,32^ these properties can also be tuned, for example, by twisting constituent layers.^28,33,34^ Furthermore, formation of interlayer excitons has also been reported in bulk MoTe_2_.^30^ While engineering type II band alignments in heterostructures, including layered perovskites, has become increasingly possible, thanks to the rapid development of scalable synthesis techniques,^35,36^ it is not clear how interlayer electronic coupling might be achieved in these systems given that the organic molecules can separate metal halide layers sometimes by very large distances (on the order of nanometers).^37^ Therefore, a microscopic understanding of the extent to which excitons might be able to delocalize across layers in bulk layered organic–inorganic perovskites is required, and first-principles calculations play a key role in this context. In this Letter, we start to develop this understanding through a detailed first-principles study of the spatial delocalization of excitons in bulk layered perovskites.

The *GW* approximation^38^ and the Bethe–Salpeter equation^39,40^ (*GW*+BSE) are state-of-the-art methodological frameworks for computing the excited state properties of semiconductors and insulators and have been applied successfully to understand the photophysics of heterogeneous systems, including TMDCs^41,42^ and metal halide perovskites.^43−48^ Spatial delocalization of excitons has been revealed in complex materials systems such as the bulk^30^ and heterostructures^33^ of TMDCs by visualizing the two-particle exciton wave function computed as a solution of the BSE. Furthermore, the computed exciton wave function can be used to estimate the average electron–hole separation corresponding to a particular excited state and to quantitatively assess the real space extent of the exciton wave function, as was shown, for example, in organic semiconductors.^49,50^ Because of the structural and chemical complexity of organic–inorganic layered halide perovskites, only a few first-principles studies of optical excitations have been reported in the literature for these systems,^17,18,51,52^ which focus on computing the quasiparticle band structure and optical absorption spectra.

In this Letter, we present for the first time a detailed analysis of the exciton delocalization in bulk organic–inorganic layered perovskites. We identify key structural features in layered perovskites that primarily determine the distribution of photoexcited electron–hole pairs across these materials. Moreover, we quantify the tunability of the interlayer delocalization of excitons through control of the distance and alignment of the inorganic layers via the organic molecular spacers. Using state-of-the-art *GW*+BSE, we rationalize the physical mechanism for interlayer delocalization of excitons to be based on the orbital decomposition of band edge states. Our study focuses specifically on lead iodide layered perovskites with planar inorganic layers that are one octahedron thick (depicted schematically in Figure 1a–c), where quantum confinement effects are strongest.^51^

**Figure 1:**
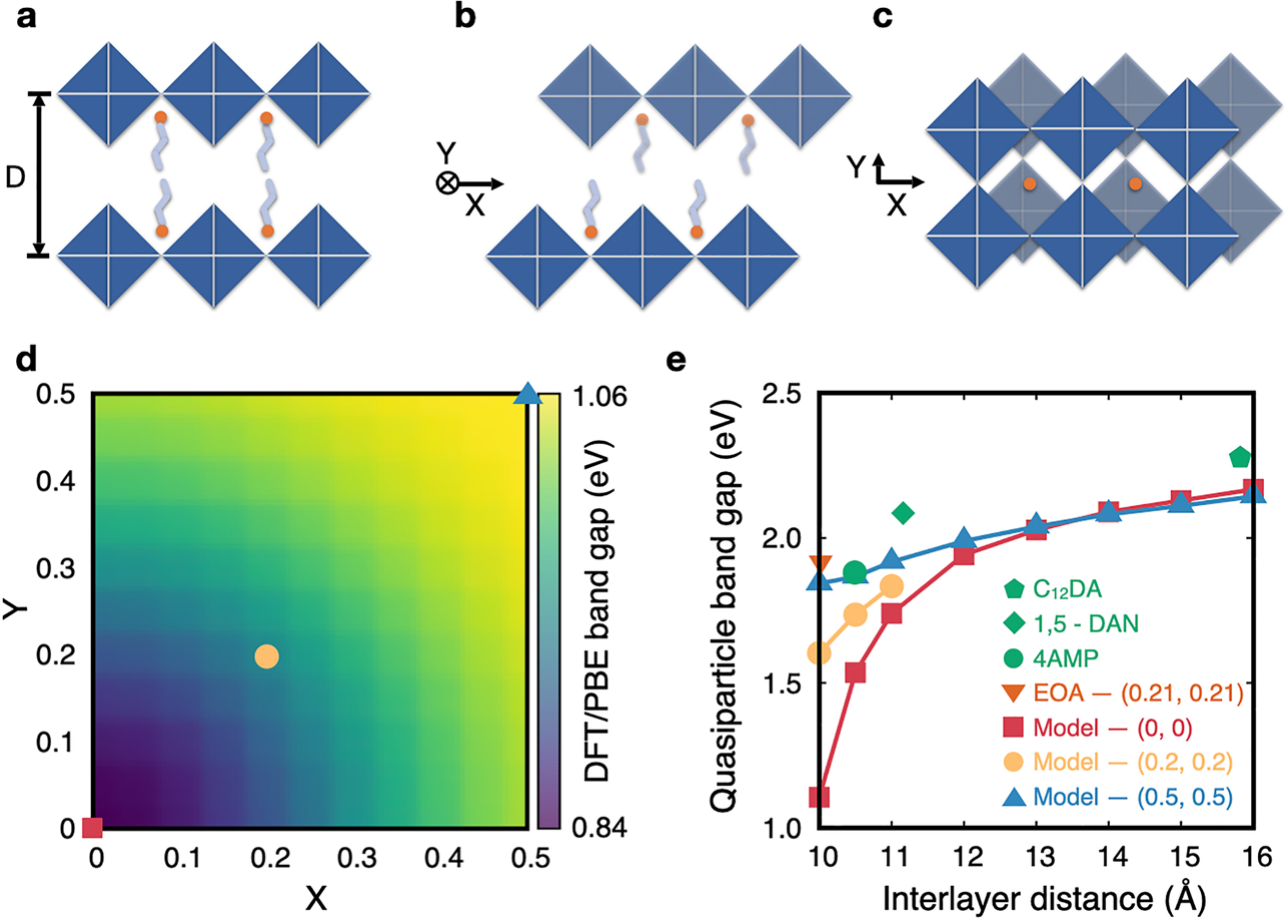
Schematic representation of (a) Dion–Jacobson and (b) Ruddlesden–Popper models viewed along the inorganic layer and (c) an intermediate phase along the direction perpendicular to the inorganic layer. Interlayer distance *D* and alignment coordinates (*X*, *Y*) are represented in panels a–c. Alignment coordinates *X* and *Y* are defined in crystal coordinates and correspond to the in-plane projection of the vector connecting two closest Pb atoms from adjacent inorganic layers. (d) DFT/PBE band gaps for model layered perovskites with an interlayer distance of 11 Å, as a function of alignment coordinates *X* and *Y*. (e) Quasiparticle band gaps of layered perovskites as a function of interlayer distance and layer alignment for different structure types: RP models (blue triangles), intermediate models (yellow disks), and DJ models (red squares). Green data points with different shapes correspond to experimental structures in DJ alignment, and the orange triangle corresponds to an experimental structure with intermediate (0.21, 0.21) alignment.

We start by investigating how the separation distance between adjacent inorganic layers (hereafter termed interlayer distance *D*) and the alignment between two adjacent layers, (*X*, *Y*) (see Figure 1a–c), impact the fundamental band gaps of layered perovskites. To this end, we construct a set of model layered perovskite structures, with undistorted inorganic PbI_6_ octahedra, and organic cations replaced by Cs (see section S1 of the Supporting Information for the details of model construction) and compute their electronic structure within density functional theory (DFT)^53,54^ and the *GW* approximation^38,55,56^ (see section S2 of the Supporting Information for computational details and convergence tests). Such model systems have been successfully used by us and others^17,18,57,58^ to capture the main trends in the photophysics of layered perovskites at a reduced computational cost. The bottom left and top right corners of the map shown in Figure 1d correspond to the two usual classifications of layered perovskites, namely, the Dion–Jacobson (DJ) phase^3,59^ with alignment (0, 0) and the Ruddlesden–Popper (RP) phase^60−62^ with alignment (0.5, 0.5). Figure 1d shows computed band gaps within DFT based on the generalized gradient approximation,^63^ including spin–orbit coupling (DFT/PBE+SOC), which exhibit a blue shift as the layer alignment changes from DJ toward RP, consistent with prior DFT studies in the literature.^64^ Second, we compute *G*_0_*W*_0_ quasiparticle band gaps (see section S2 of the Supporting Information for details) for a subset of the structures analyzed in Figure 1d with different interlayer distances. As shown in Figure 1e, quasiparticle band gaps are strongly dependent on both the interlayer distance and alignment, with values of 0.3 and 1.0 eV for RP and DJ perovskites, respectively. A similar trend is seen in band gaps computed within standard DFT/PBE (see Figure S1), indicating that this dependence is predominantly dictated by the changes in the crystal structure geometry. At large interlayer distances, DJ and RP perovskites yield nearly identical band gaps, consistent with the expectation that the amount of vacuum between the layers is converging toward the monolayer limit. A layer alignment of (0.2, 0.2) (hereafter termed intermediate) yields a similar quasiparticle band gap trend.

In Figure 1e, we corroborate these trends by computing quasiparticle band gaps for four structures of experimentally realized layered perovskites, namely, (4AMP)PbI_4_ (4AMP = 4-aminomethyl piperidinium),^59^ (C_12_DA)PbI_4_ (C_12_DA = 1,12-dodecane diammonium),^65^ (1,5-DAN)PbI_4_ (1,5-DAN = naphthalene-1,5-diamine),^65^ and (EOA)PbI_4_ (EOA = ethanolammonium),^66^ which sample DJ alignment with interlayer distances of 10.5, 11.2, and 15.8 Å and intermediate alignment (0.21, 0.21) with an interlayer distance of 10.0 Å, respectively. Experimental and model structures outline the same trend with quasiparticle band gaps of experimental structures being slightly larger than those corresponding to models. We attribute the agreement with band gaps computed for the models to a cancellation of errors originating from the absence of both octahedral tilting and organic cations in the model structures. The former is expected to red-shift quasiparticle band gaps by >0.5 eV^17,67^ (as shown in Figure S1), while the latter has been shown to blue-shift quasiparticle band gaps by >0.3 eV due to an underestimation of dielectric screening.^17,52^ Furthermore, we expect all our computed quasiparticle band gaps to be underestimated with respect to experiment by approximately 0.5 eV, due to the DFT starting point sensitivity of *G*_0_*W*_0_ calculations,^68^ and on the basis of prior studies of excited states for halide perovskites^17,43,45,52,69^ (see section S2 of the Supporting Information). Taking into account these subtleties, the agreement between band gap trends computed for model and experimental structures supports our starting assumption that calculations on model layered perovskites should capture the principal physical trends and, therefore, can be used to further explore excited state properties in a systematic way.

To rationalize the band gap trends shown in Figure 1, we analyze the quasiparticle band structures for model perovskites with DJ alignment and varying interlayer distances and for one with intermediate alignment (0.2, 0.2) and an interlayer distance of 10 Å (Figure 2a) (see Figure S3 for representative extended band structures). In addition to the red-shift in the quasiparticle band gap with shorter interlayer distances reported above, we also observe an increase in the dispersion of the valence band edge along the high-symmetry A [(0.5, 0.5, 0.5)]–M [(0.5, 0.5, 0)] direction (in reciprocal lattice units), which corresponds in real space to the direction perpendicular to the inorganic layer. The A–R [(0, 0.5, 0.5)] direction, parallel to the inorganic layer in real space, displays the opposite trend. At the same time, the conduction band edge along the A–M direction follows a similar yet more gradual change in band curvature than the valence band edge, while the A–R direction remains unaffected by the interlayer distance or alignment. These observations are consistent with the calculated charge carrier effective masses shown in Table S3 and can be explained from the analysis of the orbital contribution at the conduction and valence band edges (Figure 2b,c). The electronic wave function corresponding to the valence band top (VBT) is renormalized as the distance between the inorganic layers decreases. At large interlayer distances and/or in RP models, the VBT is degenerate at the A and M points and consists of a predominant I-p character, with apical (out-of-plane) and equatorial (in-plane) I-p orbitals contributing almost equally. As the interlayer distance decreases and the alignment approaches DJ, the A/M degeneracy splits and the VBT at the A point has a predominant contribution from apical I-p orbitals. The overlap of the adjacent out-of-plane I-p orbitals along the direction perpendicular to the inorganic layer increases, yielding disperse electronic bands along the corresponding reciprocal space path. These trends are consistent with prior reports of band structures in similar layered perovskites^64^ and suggest that by tuning the interlayer distance and alignment it is possible to induce interlayer electronic coupling in otherwise quantum-confined layered perovskites. Similar to the band gap analysis of Figure 1, quasiparticle band structures and effective masses (Figure S4 and Table S3) computed for selected experimental structures confirm the trends extracted from model layered perovskites, indicating that the interlayer distance and alignment are the primary geometric parameters that can facilitate interlayer electronic coupling, while small octahedral distortions and tilting may have a secondary effect.

**Figure 2:**
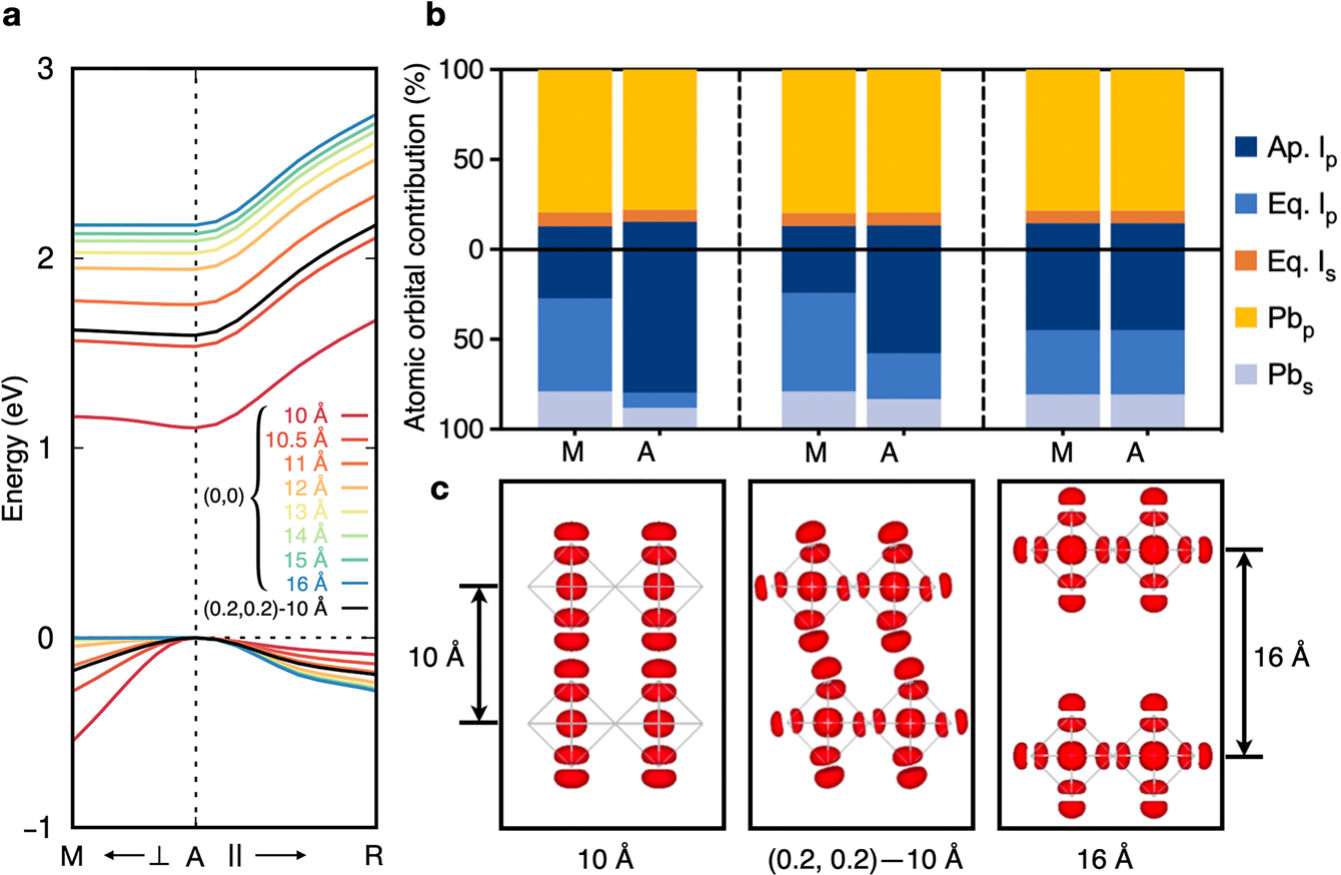
(a) Quasiparticle band structures calculated from *G*_0_*W*_0_@PBE+SOC for DJ model perovskites with interlayer distances from 10 to 16 Å and for one intermediate model with alignment (0.2, 0.2) and an interlayer distance of 10 Å. (b) Atomic orbital contribution for the VBT (bottom half) and CBB (top half) of DJ model perovskites with interlayer distances of 10 and 16 Å (left and right, respectively) and one intermediate model with alignment (0.2, 0.2) and an interlayer distance of 10 Å (middle). (c) Squared modulus of the electron wave function corresponding to the VBT at high symmetry point A for DJ layered perovskites with interlayer distances of 10 and 16 Å (left and right, respectively) and an intermediate model with alignment (0.2, 0.2) and an interlayer distance of 10 Å (middle).

Linear optical absorption spectra computed within the *GW*+BSE framework (see section S2 of the Supporting Information for computational details) for model layered perovskites are shown in panels a and b of Figure 3 and Figures S6 and S7 and display an expected red-shift with a decrease in the interlayer distance (in agreement with experimental measurements reported in ref (70)) and for structures approaching DJ alignment. Furthermore, the line shape of the optical absorption spectrum we compute for (4AMP)PbI_4_ (Figure S9) shows very good agreement with measurements reported in ref (70). In all cases, we note the emergence of a sharp peak at the onset of absorption, consistent with a bound exciton; this peak is followed by a flat plateau and a sharp rise associated with the second lowest direct optical transition. For structures with a small interlayer distance and nearly DJ layer alignment, the absorption onset red-shifts and the absorption of light polarized perpendicularly to the inorganic layers is enhanced, while the absorption of light polarized along the inorganic layers is suppressed. This observation is consistent with the renormalization of the VBT orbitals as the inorganic layers are brought sufficiently close. The signature excitonic peak also shifts closer to the “continuum” part of the spectrum as the interlayer distance decreases, suggesting that the exciton binding energy may decrease as inorganic layers are in closer proximity. We confirm this through the explicit calculations shown in Figure 3c. The range of exciton binding energies spanned for interlayer distances between 10 and 16 Å is broadest for DJ alignments, and exciton binding energies computed for experimental crystal structures closely align with calculations for model systems (Figure 3c). This is once again consistent with the assumption that the interlayer distance and alignment are the leading parameters driving these trends.

**Figure 3:**
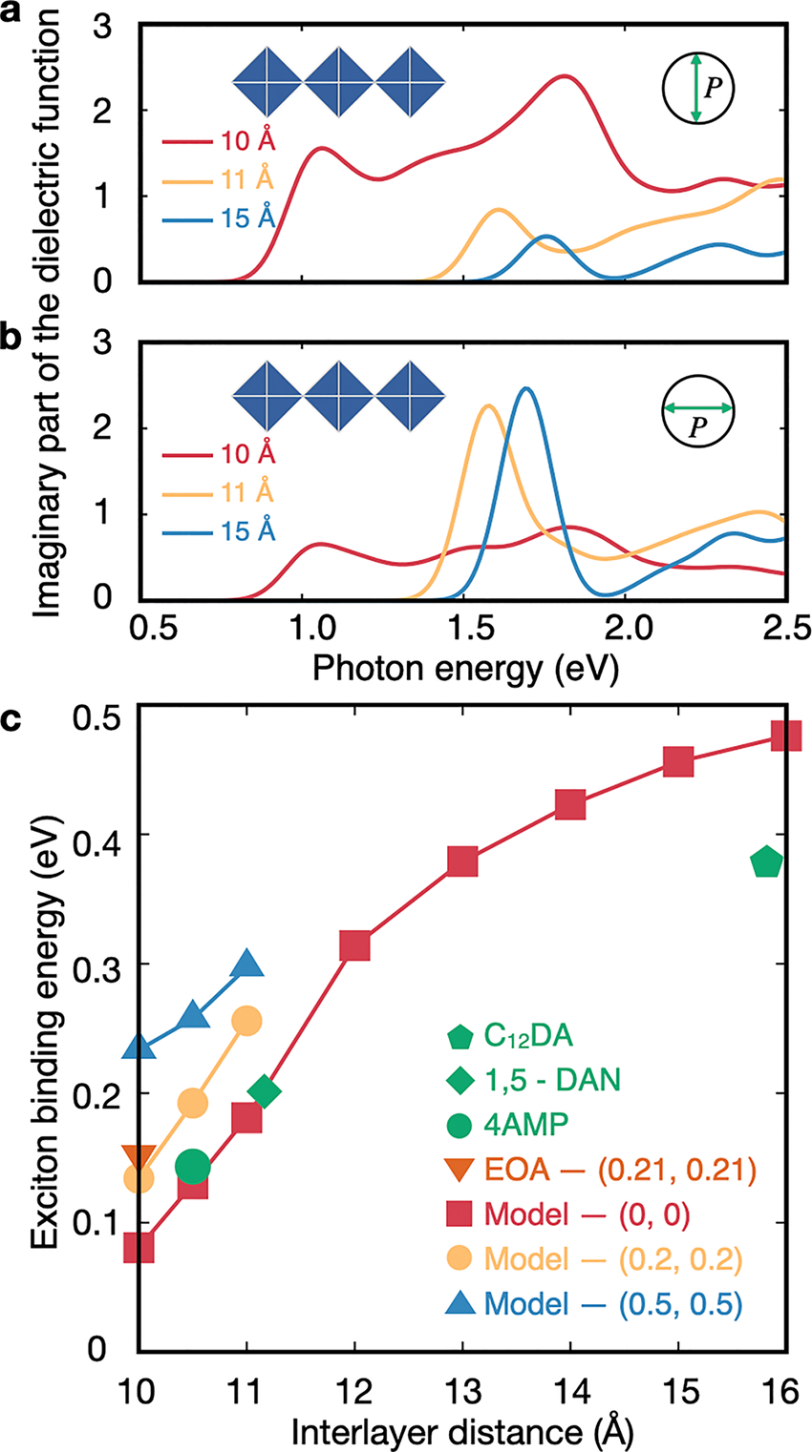
(a and b) Calculated imaginary part of the dielectric function for light polarization perpendicular to the inorganic layer and parallel to the inorganic layer, respectively, for layered perovskites with interlayer distances of 10 Å (red), 11 Å (yellow), and 15 Å (blue). Similar plots for different layer alignments and for experimental structures are reported in Figures S6 and S7. (c) Exciton binding energies computed from *G*_0_*W*_0_+BSE as a function of interlayer distance and layer alignment. The legend follows the same convention as in Figure 1e.

The exciton binding energy is generally correlated in isotropic materials with the average real space separation between photoexcited electrons and holes.^71^ The larger the energy, the smaller the electron–hole separation. On the basis of this intuition and the results shown in Figure 3c, we might expect that excitons will be more delocalized in layered perovskites with inorganic layers closer together. To probe if this intuitive picture is valid here, we analyze the two-particle exciton wave function, Ψ(**r**_e_, **r**_h_), corresponding to the lowest (nondegenerate) bound state, where **r**_e_ and **r**_h_ are position vectors for the electron and hole, respectively. First, we visualize the probability of localization for a photoexcited electron when the hole is fixed in an arbitrary position, shown in Figure 4 and Figure S8 for two different interlayer distances. We find that photoexcited electrons are strongly confined within a single inorganic layer for the larger interlayer distance of 16 Å, in agreement with prior works^17,18^ (see Figure S8). In contrast, Figure 4a shows a nontrivial probability of localization for photoexcited electrons extending across the first and second nearest neighboring layers for the smaller interlayer distance of 10.5 Å.

**Figure 4:**
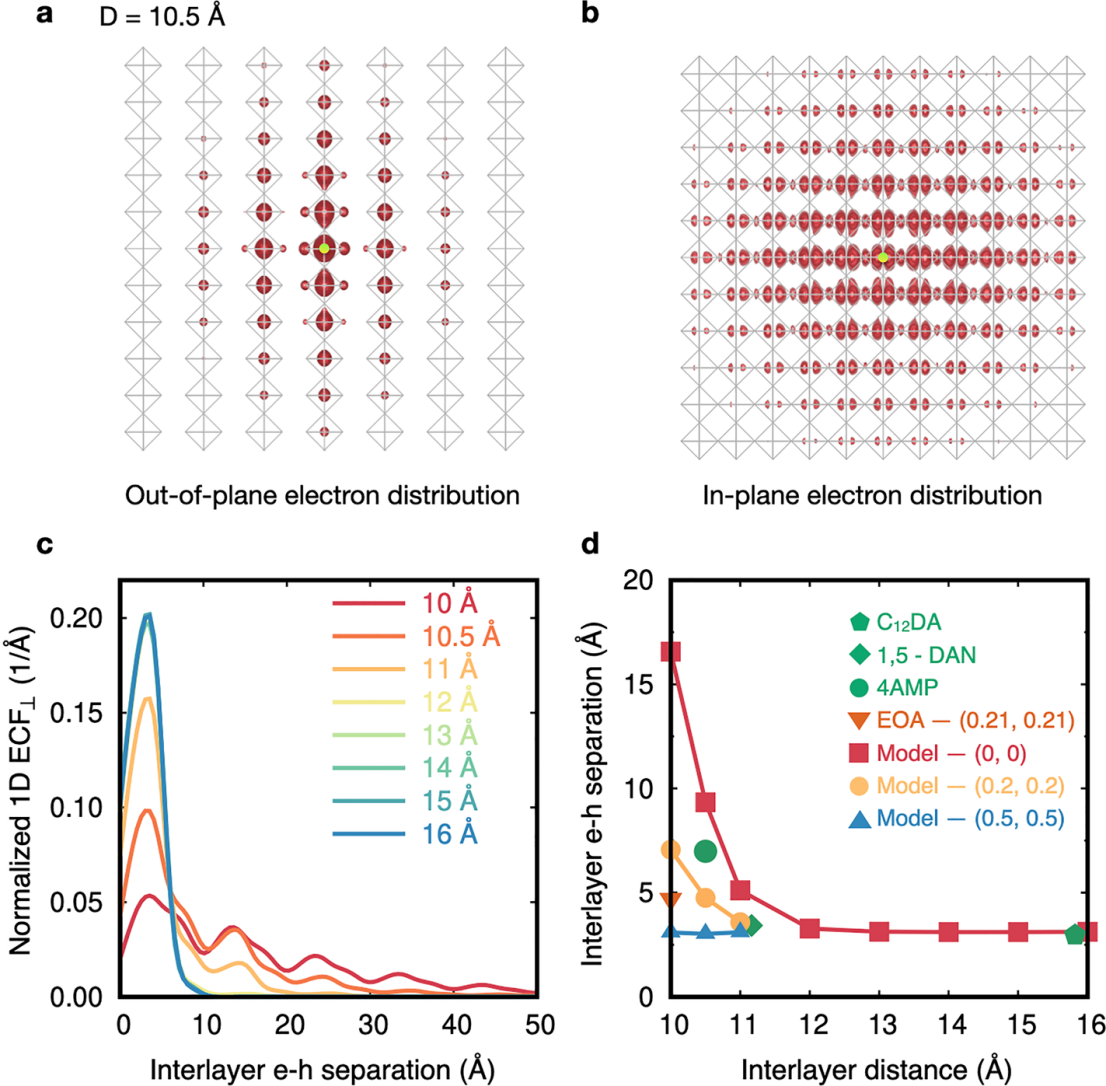
(a and b) Isosurfaces representing the out-of-plane and in-plane spatial distribution of the lowest-energy exciton for a model DJ structure with an interlayer distance of 10.5 Å. The hole position is fixed at the center Pb atom of the central layer, marked by the green points. A similar diagram is shown for a model DJ structure with an interlayer distance of 16 Å in Figure S8. In both (a) and (b) the Cs ions are removed for clarity and the lead-halide octahedra are represented by the grey squares. (c) Normalized one-dimensional ECF vs electron–hole relative position across layers, for DJ model structures with interlayer distances from 10 to 16 Å. (d) Average interlayer electron–hole separation as a function of the interlayer distance and layer alignment. The legend follows the same convention as in Figure 1e.

While Figure 4 and Figure S8 may suggest that exciton delocalization can be tuned by changing the interlayer distance in layered perovskites, it does not provide a quantitative assessment of this tuning vehicle. To compute this, we analyze the exciton correlation function (ECF), as introduced in ref (49) and defined as , where **r** = **r**_e_ – **r**_h_ and Ω_uc_ and Ω are the volumes of the primitive unit cell and a supercell large enough to contain the full extent of the exciton, respectively. The ECF is the probability that the electron and hole in a specific state are separated by the vector **r**(49,72) (see section S4 of the Supporting Information for details); the main advantage of evaluating this quantity is that it is independent of the arbitrary choice of hole positions, in contrast with the qualitative pictures shown in panels a and b of Figure 4 and Figure S8.

We use the ECF to quantify how extended the lowest-energy bound exciton is in layered perovskites and specifically analyze it in plane as  and out of plane as , where the *z* direction is the direction normal to the plane. As expected, the in-plane one-dimensional (1D) ECF_∥_ calculated for model DJ structures (shown in Figure S11) displays a wider extent for smaller interlayer distances. For all structures analyzed (Figure 4c and Figure S12), the out-of-plane 1D ECF_⊥_ displays a narrow peak centered around 3.1 Å away from the origin, indicating that bound electron–hole pairs in these states are most likely to be 3.1 Å apart (approximately the width of a Pb–I bond length or half the width of one inorganic layer). However, while the height of this peak is nearly 0.2 for model layered perovskites with a large interlayer distance and/or RP alignment, it decreases sharply to ≤0.05 when the inorganic layers are DJ aligned and 10 Å apart (see Figure 4c and Figure S12). In the latter case, in addition to the main ECF peak, several equally spaced peaks are clearly distinguished for larger electron–hole distances, consistent with an interlayer delocalization of the exciton wave function (Figure 4c and Figure S12). We quantify the average interlayer electron–hole separation as described in section S4 of the Supporting Information and shown in Figure 4d for all model layered perovskites with different interlayer distances and alignments and for all representative experimental structures. We find that the average interlayer electron–hole separation decreases from >16 Å (interlayer delocalized) to ∼3 Å (intralayer localized), as interlayer distance *D* increases from 10 to 12 Å for DJ aligned perovskites, with a slower decrease for the intermediate aligned perovskites. Beyond an interlayer distance of 12 Å, the average interlayer electron–hole separation remains roughly constant. In contrast, lowest-energy excitons are confined within a single metal halide layer in all RP aligned structures, regardless of their interlayer distance.

Overall, our results demonstrate that exciton delocalization across the inorganic metal halide layers in is primarily driven by the electronic coupling between neighboring apical halogen p orbitals. In sufficiently close proximity, the apical I-p orbitals dominating the VBT overlap with one another and with the CBB wave functions and yield non-negligible contributions to the lowest-energy exciton wave function from pairs of single-particle states localized in separate inorganic layers. Excitons delocalized across layers correspond to states with a low excitation energy and a low exciton binding energy. Furthermore, due to the interlayer electronic coupling, perovskites with small interlayer distances exhibit absorption coefficients with similar magnitudes for both in- and out-of-plane polarized light.

In summary, we have performed state-of-the-art *GW*+BSE calculations for a series of model and experimental structures of layered organic–inorganic lead iodide perovskites to understand how the distance and alignment of inorganic layers impact the excited state properties of this family of materials. We found that electronic and optical coupling of adjacent inorganic layers can be achieved by tuning the interlayer distance and relative layer alignment and is facilitated through the interaction of apical I orbitals from neighboring inorganic layers. We have shown for the first time that through interlayer coupling, it is possible to overcome the confinement of excitons in a single lead iodide layer and facilitate the interlayer delocalization of bound electron–hole pairs in bulk layered perovskites. The interlayer distance in layered materials and heterostructures could be controlled in principle through applied pressure.^73,74^ However, strain-induced structural changes in both the inorganic and the organic layers may be more difficult to control in heterogeneous and soft layered perovskites and may have secondary effects on the electronic structure.^74^ Unlike conventional layered materials, tuning interlayer separation and alignment in layered perovskites can also be realized intrinsically, through a judicious choice of the organic spacers.^75^ Here, we have shown that all physical trends extracted for model systems are confirmed by explicit calculations for experimentally realized layered perovskites chosen as representatives of each structural feature. We hope that our work will provide a reliable starting point for future studies that aim to understand the role of phonons in the localization of excitons, in these complex systems, using for example similar first-principles approaches to those recently reported in ref (50). Additionally, the intuition derived here may also be transferred to isolated nanostructures, including a bilayer or multiple exfoliated layers^76^ and self-assembled bulk heterostructures,^77^ providing a potential pathway toward the exploration of layered organic–inorganic halide perovskites as functional materials for possible applications in excitonic devices.

## Supporting Information Available

Computational and methodological details, tables, and plots, including convergence tests (PDF)
