# Supplementary material for: Tunable Interlayer Delocalization of Excitons in Layered Organic–Inorganic Halide Perovskites
Source: J Phys Chem Lett. 2023 Nov 20;14(47):10634–41. doi: 10.1021/acs.jpclett.3c02339 (PMC10694835; doi:10.1021/acs.jpclett.3c02339)
Supplement: Supplementary file 1 — jz3c02339_si_001.pdf [file jz3c02339_si_001.pdf]

Tunable Interlayer Delocalization of Excitons in Layered Organic-Inorganic Halide Perovskites Supplementary Information

Yinan Chen and Marina R. Filip*

Department of Physics, University of Oxford, Clarendon Laboratory, Oxford OX1 3PU, U.K.

E-mail: marina.filip@physics.ox.ac.uk

S1 Model construction

In this work, we construct a set of model layered perovskite in a similar approach to that used in Ref. 1 in which the organic cation is replaced by a monovalent Cs to obtain Cs_2PbI_4. These are referred to across the main manuscript and SI as 'model structures' (DJ, RP or intermediate). The unit cell of each model structure consists of a sheet of corner-shared undistorted and untilted PbI_6 octahedra, with the Pb-I bond length being fixed at 3.184 Å. This bond length value was obtained from the structural optimization of a 3D $CsPbI_3$ perovskite in the cubic phase, using density functional theory (DFT)[2] and the PBE functional. The interlayer distance is defined as the distance between inorganic layers as measured from the plane including all Pb atoms at the center of the octahedra. In our models, we vary the interlayer distance from 10 Å to 16 Å with a step of 1 Å. An additional interlayer distance of 10.5 Å is also included in order to match the lowest interlayer distance of the Dion-Jacobson experimental structures in our study. In each case, we obtain the model structures

by relaxing the Cs atoms, while keeping the PbI_6 layers and lattice parameters fixed.

S2 Computational setup

S2.1 Ground state calculations

DFT calculations carried out in this work were performed using the Quantum Espresso package (version 6.7).[3,4] We employed generalized gradient approximation in the Perder-Burke-Ernzerhof parametrization[5] to compute the exchange-correlation potential and used fully relativistic, norm-conserving ('standard') pseudopotentials as reported in the Pseudo Dojo repository.[6] We included spin-orbit coupling (SOC) effects in all calculations of electronic structure and optical properties. We use a kinetic energy cutoff of 50 Ry and $\mathbf{k}$ points are sampled using the Monkhorst-Pack scheme with a Γ centered $6 \times 6 \times 4$ mesh, where the sparser sampling is applied in the direction perpendicular to the perovskite planes.

S2.2 Excited state calculations

S2.2.1 Quasiparticle band structures

We compute the quasiparticle band structures of all systems studied in this manuscript within a single-shot G_0W_0 approximation, as implemented in the BerkeleyGW code.[7] We calculate quasiparticle energies $E_{n\mathbf{k}}$, starting from mean-field DFT/PBE+SOC eigenvalues $\epsilon_{n\mathbf{k}}$ as:

$$E_{n\mathbf{k}} = \epsilon_{n\mathbf{k}} + Z_{n\mathbf{k}}\left(\epsilon_{n\mathbf{k}}\right)\left\langle n\mathbf{k}\left|\Sigma\left(\epsilon_{n\mathbf{k}}\right) - V_{\mathrm{xc}}\right| n\mathbf{k}\right\rangle \tag{1}$$

where V_{xc} is the exchange-correlation potential, and $Z(\omega) = [1 - \mathrm{Re}(\partial\Sigma/\partial\omega)]^{-1}$ is the quasiparticle renormalization factor, $\Sigma(\omega)$ is the electron self energy computed within the G_0W_0 approximation as the convolution of the screened Coulomb interaction W_0 and the single-particle Green's function G_0, schematically written as $\Sigma = iG_0W_0$. We include spin-orbit coupling in all quasiparticle band structure calculations. We compute the non-local, fre-

quency dependent dielectric function within the random phase approximation, using the Godby-Needs plasmon-pole model,[8] as implemented in the BerkeleyGW code.[7] Calculation parameters for all cases studied (informed by convergence tests in Figure S2, and similar to convergence parameters used in Ref. 1) are listed in Table S1.

This setup achieves quasiparticle band gaps which are converged within approximately 0.1-0.2 eV. We note that quasiparticle band structures computed in this setup are expected to be underestimated with respect to experiment by approximately 0.5 eV. This is due to the sensitivity of single-shot G_0W_0 calculations to the mean-field starting point, which was pointed out for halide perovskites and other seminconductors in several recent reports.[1,9–11]

We compute quasiparticle band structures by interpolating from the coarse grid (see Table S1) to the full Brillouin zone path along the high-symmetry points using wavefunction projections, as implemented in the BerkeleyGW code (inteqp.x utility). Figure S3 shows full quasiparticle band structures for two representative Dion-Jacobson perovskite models with different interlayer distances (D = 10.5 Å and D = 15 Å, respectively).

S2.2.1 Optical absorption spectra and exciton binding energies

We compute the two-particle excitation energies by solving the Bethe-Salpeter equation (BSE), within the Tamm-Dancoff approximation,[12,13] as implemented in the BerkeleyGW code,[7]

$$\left(E^{\mathrm{QP}}_{c\mathbf{k}} - E^{\mathrm{QP}}_{v\mathbf{k}}\right) A^S_{vc\mathbf{k}} + \sum_{v'c'\mathbf{k}'} \left\langle vc\mathbf{k} \left| K^{\mathrm{eh}} \right| v'c'\mathbf{k}' \right\rangle A^S_{vc\mathbf{k}} = \Omega^S A^S_{vc\mathbf{k}} \tag{2}$$

where $A^S_{vc\mathbf{k}}$ are the coefficients of the exciton wavefunction written in the single-particle basis, Ω^S is the excitation energy, and K^{eh} is the electron-hole interaction kernel, which includes the contribution from the exchange and direct electron-hole interaction.[12] We find that exchange interaction changes the energy of the lowest excited state in $(4\,\mathrm{AMP})\mathrm{PbI}_4$ by less than 30 meV, and leads to four lowest energy non-degenerate excited states. In this work, we focus on the lowest lying state in all the cases studied. The exciton binding energy reported in this paper is calculated as the difference between the quasiparticle band gap and

the energy of the first excited state, as computed from the BSE.

We use the excitation energies and two-particle wave functions to also compute the imaginary part of the dielectric function as,[7,13]

$$\varepsilon_2(\omega) = \frac{16\pi^2 e^2}{\omega^2} \sum_S |\mathbf{e} \cdot \langle 0|\mathbf{v}|S\rangle|^2 \delta\left(\omega - \Omega^S\right) \tag{3}$$

where we approximate the velocity operator as $\mathbf{v} = i\nabla$, as discussed in Ref. 7. As with the quasiparticle energies, all calculation parameters are listed in Table S1. These choices are similar to those reported in Ref. 1. They are informed by convergence tests shown in Figure S5 and Table S2, which are expected to yield exciton binding energies converged within less than 50 meV.

S3 Charge Carrier Effective Masses

We compute the charge carrier effective masses by diagonalizing the effective mass tensor obtained from the G_0W_0 quasiparticle eigenvalues as,

$$m_{ij}^{*\mathrm{e,h}} = \left[\frac{1}{\hbar^2}\frac{\partial^2 E_{\mathrm{c,v}}(\mathbf{k})}{\partial k_i \partial k_j}\right]^{-1} \tag{4}$$

where $E_{\mathrm{c,v}}(\mathbf{k})$ are the conduction band minimum and valence band maximum, respectively. Second derivatives are computed numerically, using a discretization step δk_i of 0.01 bohr^{-1}. Calculated effective masses are shown in Table S3.

S4 Exciton Correlation Function

The two-particle exciton wave function can be written using the eigenvectors $A^S_{vc\mathbf{k}}$ of the BSE Hamiltonian, within the single-particle basis as:

$$\Psi^S(\mathbf{r}_e, \mathbf{r}_h) = \sum_{cv\mathbf{k}} A^S_{vc\mathbf{k}} \psi_{c\mathbf{k}}(\mathbf{r}_e) \psi^*_{v\mathbf{k}}(\mathbf{r}_h) \tag{5}$$

where $\mathbf{r}_{e(h)}$ correspond to the position of the electron (hole), and $\psi_{c(v)\mathbf{k}}$ are the single particle wave functions corresponding to unoccupied (occupied) states. Figures 4(a,b) of the main manuscript and S8 show examples of these exciton wave functions by depicting the localization probability for a photoexcited electron in the lowest bound state, when the hole is fixed at an arbitrarily chosen position in the inorganic layer, $|\Psi(\mathbf{r}_e, \mathbf{r}_h = \text{fixed})|^2$.

In order to achieve a quantitative picture of exciton delocalization that does not depend on arbitrarily sampling the position of the photoexcited hole, we compute the electron-hole correlation function (ECF) $\mathcal{F}(\boldsymbol{r})$, introduced in Ref. 14, which describes the probability of finding electron and hole separated by a relative position vector, $\boldsymbol{r}$:

$$\mathcal{F}(\boldsymbol{r}) = \frac{\int_{\Omega_{\mathrm{uc}}} |\Psi(\boldsymbol{r}_e = \boldsymbol{r} + \boldsymbol{r}_h, \boldsymbol{r}_h)|^2 \, d\boldsymbol{r}_h}{\int_{\Omega} \int_{\Omega_{\mathrm{uc}}} |\Psi(\boldsymbol{r}_e, \boldsymbol{r}_h)|^2 \, d\boldsymbol{r}_e d\boldsymbol{r}_h} \tag{6}$$

The numerator of this expression is an integral of hole positions over the volume of the primitive cell Ω_{uc}, the denominator is an integral over hole positions in the primitive cell Ω_{uc} and electron position in a supercell Ω, normalizing the ECF. Furthermore, in order to describe the exciton localization across inorganic layers, we integrate the ECF along the in-plane directions of the supercell, to obtain the out-of-plane integrated and normalized ECF (normalized 1D $\mathrm{ECF}_\perp$ in Figure 4c),

$$\mathcal{F}_\perp(z) = \int_\Omega \mathcal{F}(\boldsymbol{r}) dx dy. \tag{7}$$

To aid comparison, we can perform this integration for any other direction, and plot corre-

sponding ECFs, as shown for example the normalized 1D ECF corresponding to an in-plane direction in Figure S11.

We compute the integral in the numerator of Eq. 6 as a discrete sum over $\boldsymbol{r}_h$, sampled with a uniform grid in the primitive unit cell. In this expression, the real space wave function $|\Psi(\boldsymbol{r}_e, \boldsymbol{r}_h)|^2$ for each sampled $\mathbf{r}_h$ is computed using the plotxct.x utility of the BerkeleyGW package using a downsampled real-space grid. The hole sampling grid density was carefully tested to achieve a converged ECF (shown in Figure S10 and Table S4). Given the convergence tests shown in Figure S10, for all systems studied we only sampled holes along one line that is perpendicular to the perovskites layer in the unit cell, with a grid spacing around 0.3 Å. This choice maintains an accuracy of less than 0.3 Å for the calculated interlayer electron-hole separation for the smallest interlayer distance model (see Table S4). For each hole position, the real space exciton wave function is computed in a 14 × 14 × 10 supercell to ensure the wavefunction decays to less than 0.1% of the maximum peak value at the boundary.

We also note that throughout this study, we perform the analysis of the wave function for the lowest energy excitonic state. This is the first in a group of four lowest energy excited states, originating from spin degenerate valence band top and conduction band bottom (discussed also in Ref. 1). It is an optically inactive state and we have specifically chosen this state instead of the subsequent optically active states because it is non-degenerate (and hence less computationally demanding to analyze). In order to test that our conclusions are not sensitive to this choice, we show the ECF displays a very similar profile for all four states in two DJ models with different interlayer distances (Figure S11) and average electron-hole separation calculated for the four different states varies within 2.2 Å for the in-plane direction and 1.6 Å for the interlayer direction (see Table S5).

We calculate the average interlayer electron-hole separation as:

$$\langle r_\perp \rangle = \int_\Omega |z| \mathcal{F}(x, y, z) dx dy dz. \tag{8}$$

and the corresponding in-plane quantity as:

$$\langle r_{\parallel} \rangle = \int_{\Omega} |x| \mathcal{F}(x, y, z) dx dy dz. \tag{9}$$

Table S1: Computational setup for GW and BSE calculations.
*The same **k**-point grid was used for the calculation of the dielectric function and quasiparticle corrections. The dielectric function was computed on a half-shifted grid, while the quasiparticle energies were calculated on a Γ-centered grid.

**Structure**	**DJ**	**RP/Intermediate**	**Exp.**
Formula unit	1	2	2
GW empty bands	400	800	800
GW plane wave cutoff	6 Ry	6 Ry	6 Ry
GW **k**-point grid*	6 × 6 × 4	6 × 6 × 2	4 × 4 × 4
e-h kernel: **k**-point grid	6 × 6 × 4	6 × 6 × 2	4 × 4 × 4
e-h kernel: val. / cond.	8 / 8	8 / 8	16 / 16
absorption **k**-point grid	30 × 30 × 10	30 × 30 × 5	20 × 20 × 10
absorption val. / cond.	2 / 2	4 / 4	8 / 8

Table S2: Calculated exciton binding energy for a model layered perovskite structure with interlayer distance of 10 Å and 1,5 - DAN using different Γ centered **k**-point grid densities and numbers of bands.

**System**	**Fine grid density**	**Val. / Cond.**	E_b **(meV)**
**DJ Model, 10 Å**	30 × 30 × 4	2 / 2	273
	30 × 30 × 6	2 / 2	248
	30 × 30 × 10	2 / 2	230
	30 × 30 × 20	2 / 2	220
	34 × 34 × 10	2 / 2	229
	30 × 30 × 10	4 / 4	231
	34 × 34 × 10	4 / 4	230
**1,5 - DAN**	20 × 20 × 10	4 / 4	184
	20 × 20 × 10	8 / 8	201
	20 × 20 × 10	12 / 12	205

Table S3: Calculated G_0W_0 electron and hole effective masses, along directions perpendicular ($\perp$) and parallel ($\|$) to the perovskite planes, in unit of electron rest mass m_0, as a function of interlayer distances D, for DJ models and experimental structures

**System**	D(Å)	**Valence** (m_h)		**Conduction** (m_e)	
		$\perp$	$\|$	$\perp$	$\|$
**DJ - Models**	10	0.10	0.41	0.33	0.13
	10.5	0.21	0.38	1.14	0.13
	11	0.42	0.33	2.54	0.13
	12	1.38	0.28	8.12	0.13
	13	4.03	0.27	24.69	0.13
	14	14.48	0.26	50.33	0.13
	15	44.45	0.26	155.78	0.13
	16	45.66	0.26	8950.98	0.13
**4AMP**	10.5	0.32	0.43	1.43	0.19
**1,5 - DAN**	11.2	1.31	0.36	2.64	0.20
**C_{12}DA**	15.8	16.51	0.26	81.42	0.20
**EOA (intermediate)**	10.0	0.59	0.29	1.73	0.16

Table S4: Calculated interlayer and in-plane average electron-hole separation for a model DJ structure with an interlayer distance of 10 Å using different hole sampling densities.

**Hole sampling density**	**Interlayer e-h sep.**	**In-plane e-h sep.**
1 × 1 × 15	17.3 Å	21.4 Å
1 × 1 × 31	17.3 Å	21.4 Å
5 × 5 × 15	17.0 Å	21.7 Å

Table S5: Calculated average electron-hole separation along the in-plane and interlayer directions for the first 4 excited states of two model DJ structures with interlayer distances of 10.5 Å and 15 Å.

**Interlayer distance**	**Excited state**	**Interlayer e-h sep.**	**In-plane e-h sep.**
10.5 Å	1	9.3 Å	10.6 Å
	2	9.8 Å	11.0 Å
	3	9.8 Å	11.0 Å
	4	10.9 Å	12.0 Å
15 Å	1	3.1 Å	6.5 Å
	2	3.1 Å	6.8 Å
	3	3.1 Å	6.8 Å
	4	3.1 Å	8.7 Å

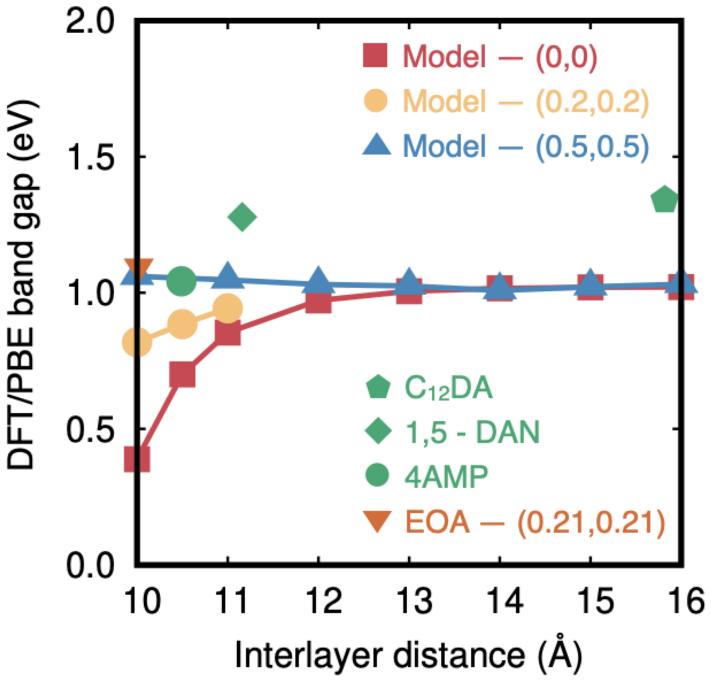

Figure S1: Band gaps of layered perovskites calculated with DFT/PBE as a function of the interlayer distance for different layer alignments. The legend for this figure is the same as that of Figure 1e of the main manuscript.

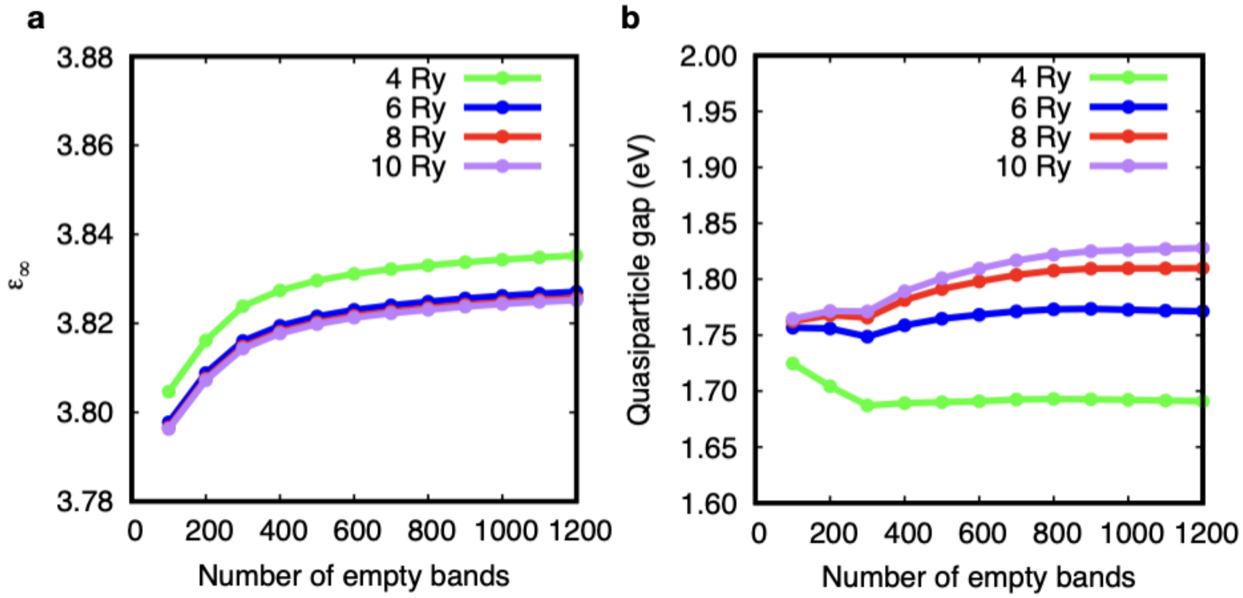

Figure S2: Convergence of (a) high frequency limit of the dielectric constants ε_∞ and (b) quasiparticle band gaps for a model DJ structure with an interlayer distance of 11 Å with respect to energy cutoff for the dielectric matrix and number of empty bands.

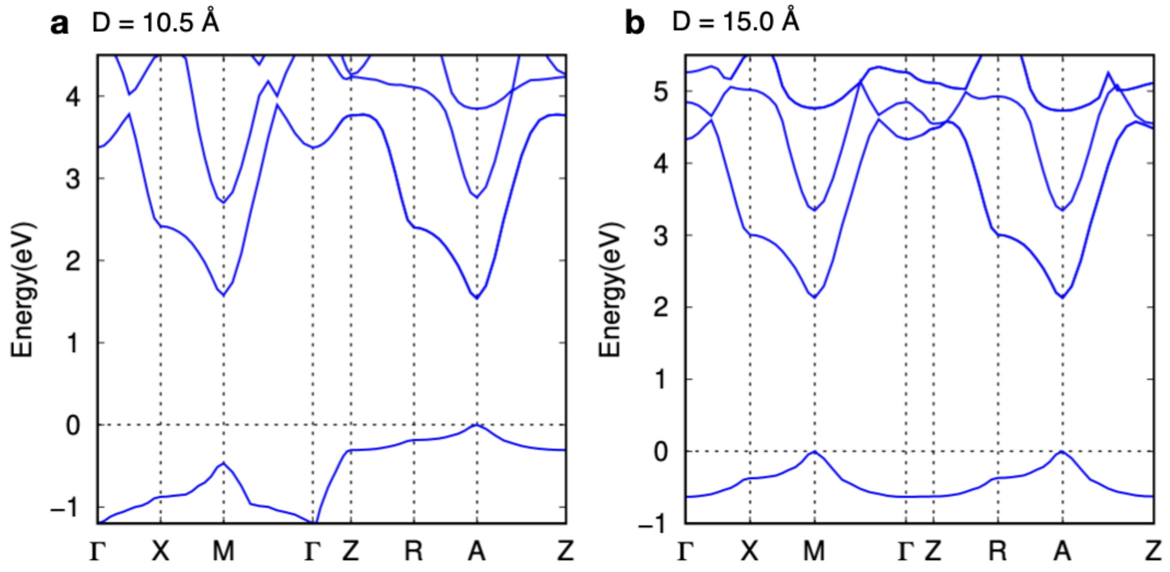

Figure S3: Quasiparticle band structures of model DJ structures with interlayer distances of 10.5 Å (a) and 15.0 Å (b).

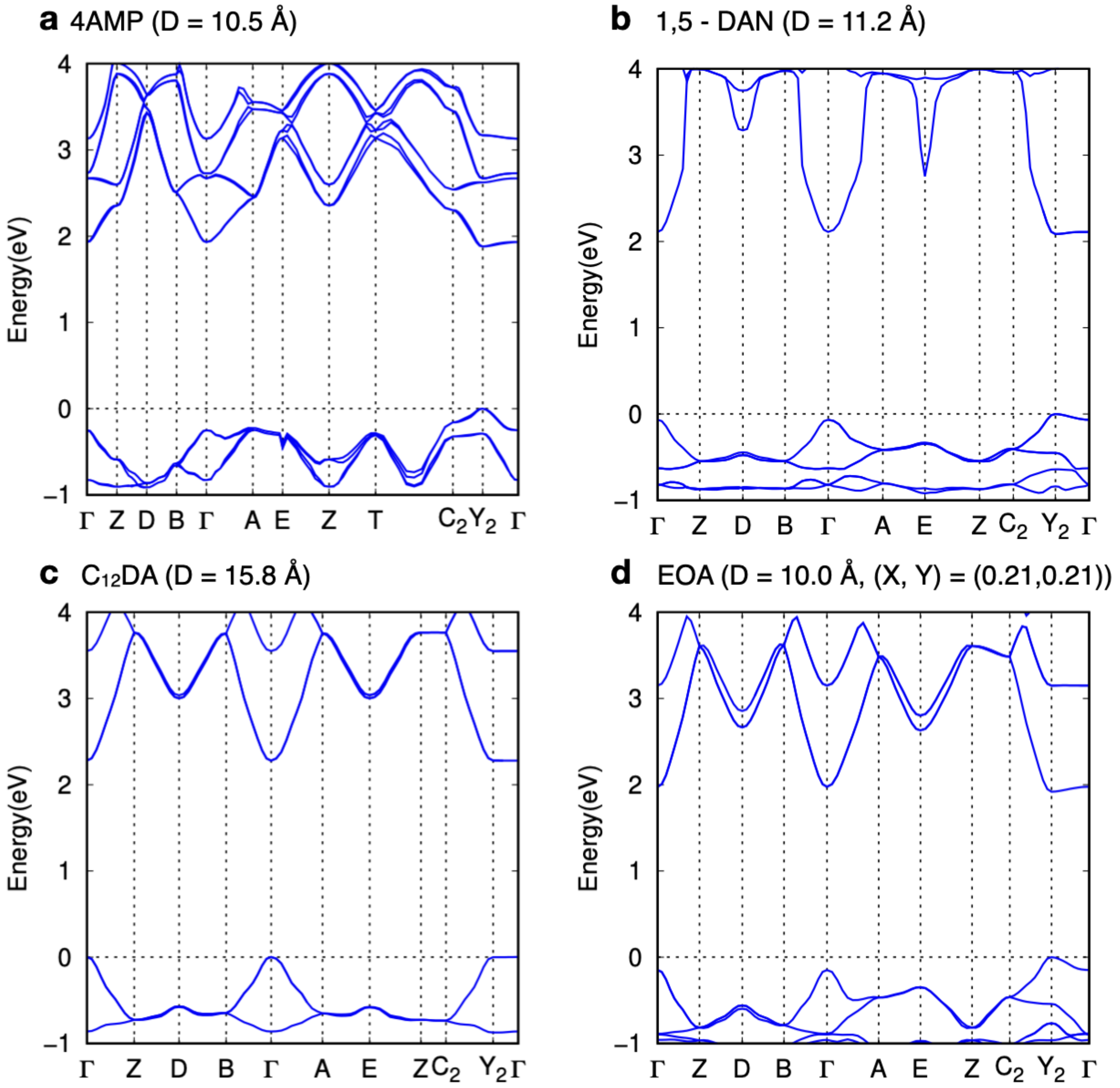

Figure S4: Quasiparticle band structures for experimental structures (a) (4AMP)PbI_4,[15] (b) (1,5 - DAN)PbI_4,[16] (c) (C_{12}DA)PbI_4[16] and (d) (EOA)PbI_4,[17] featuring different interlayer distances and different layer alignment.

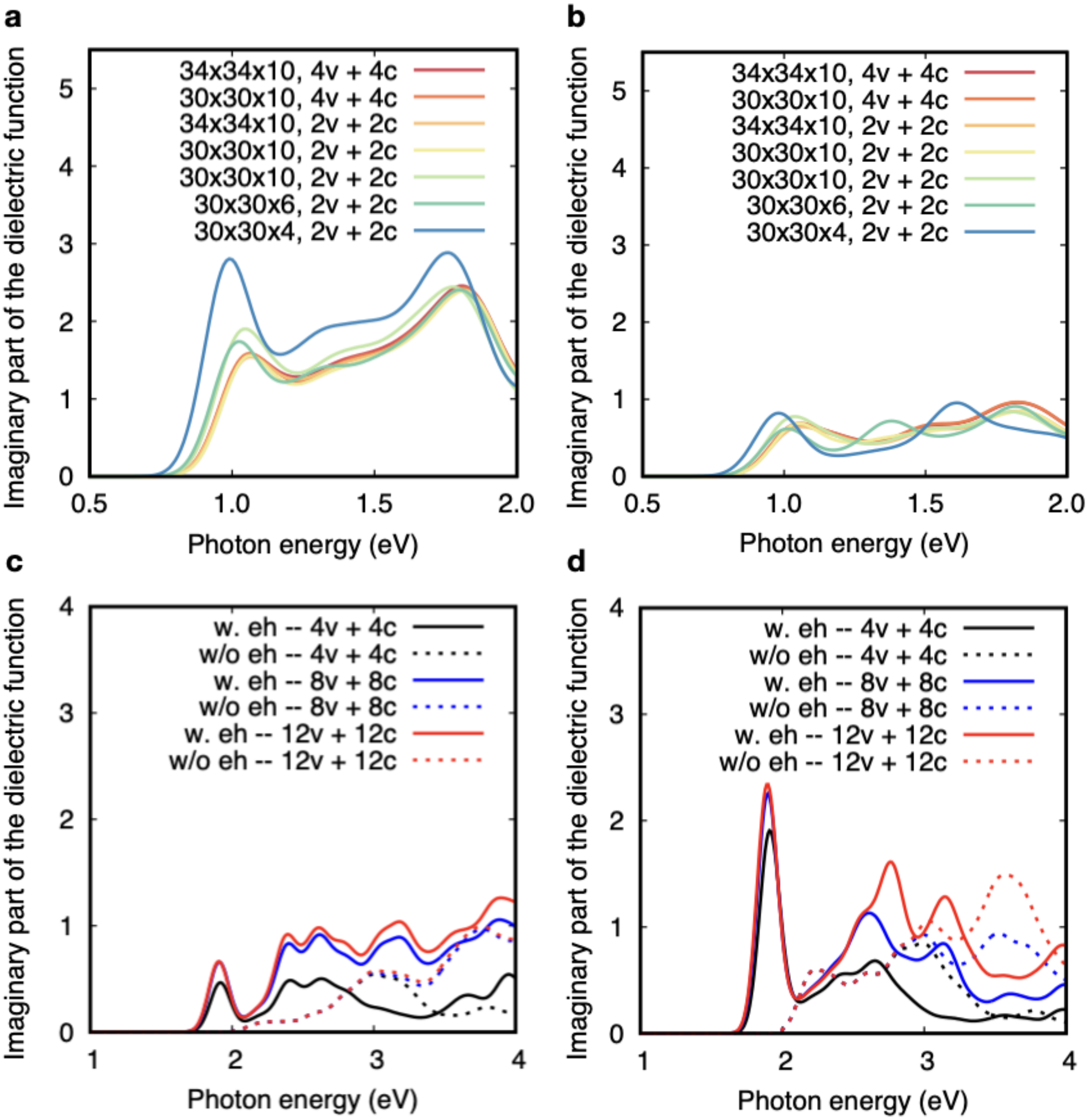

Figure S5: Calculated imaginary part of the dielectric function with light polarization perpendicular to the inorganic layer (Left) and parallel to the inorganic layer (Right) for model layered perovskites with an interlayer distance of 10 Å (a,b) using various fine grid density and numbers of bands, and for 1,5 - DAN (c, d) using a 20 × 20 × 10 fine grid and different numbers of bands.

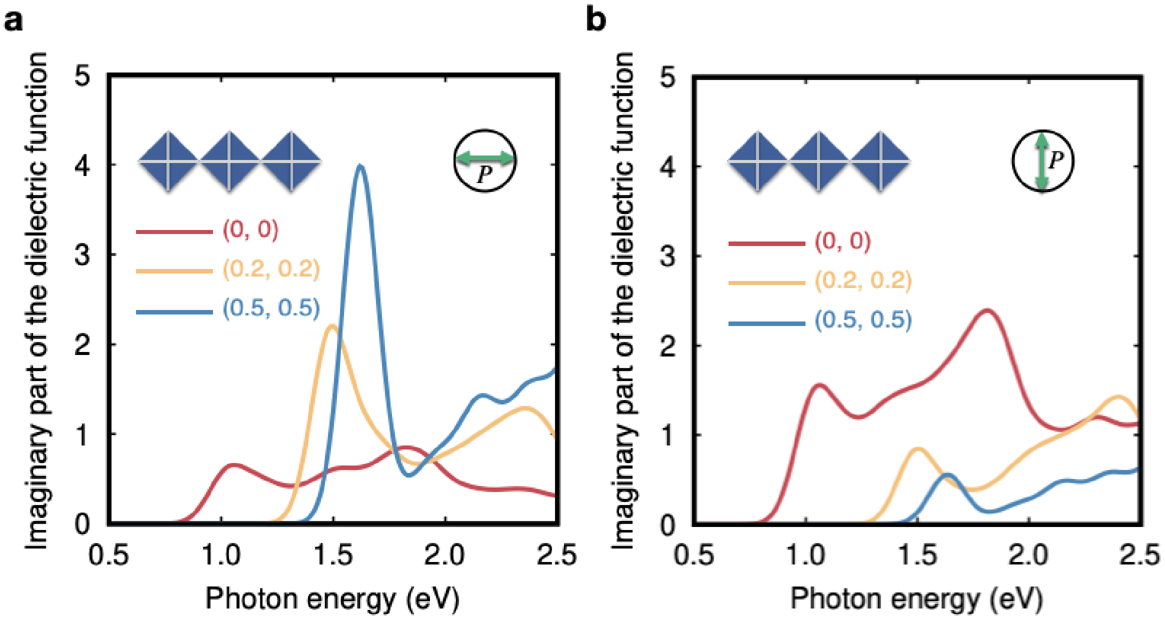

Figure S6: Calculated imaginary part of the dielectric function with light polarization parallel to the inorganic layer (a) and perpendicular to the inorganic layer (b) for model structures with different layer alignment at a fixed interlayer distances of 10 Å.

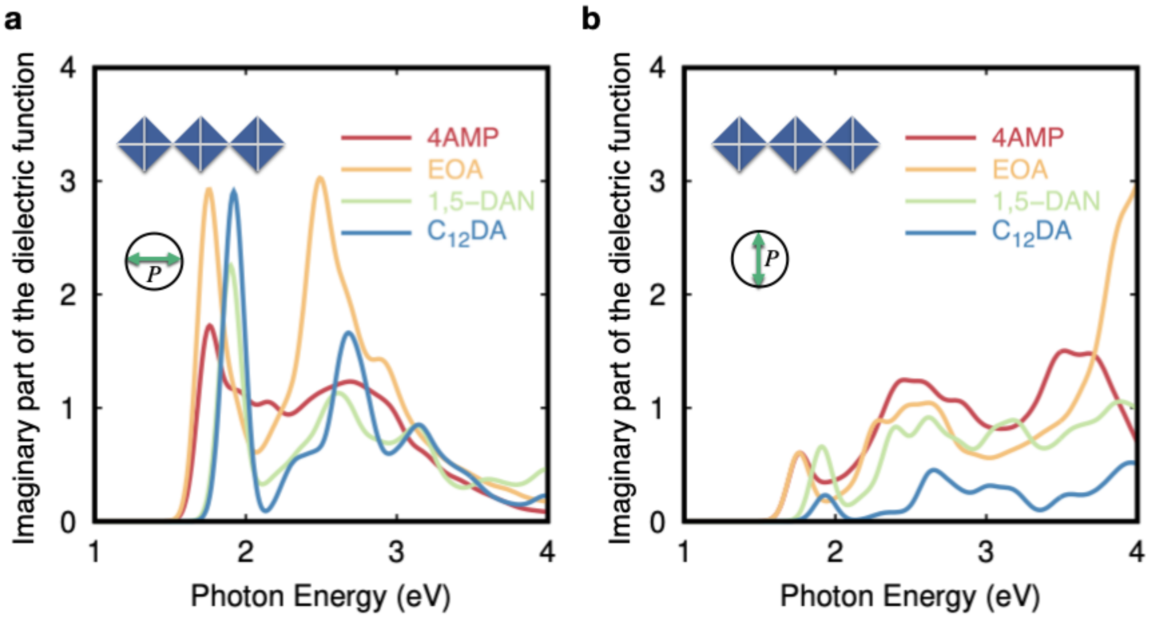

Figure S7: Calculated imaginary part of the dielectric function with light polarization parallel to the inorganic layer (a) and perpendicular to the inorganic layer (b) for four representative experimental structures as described in the main text.

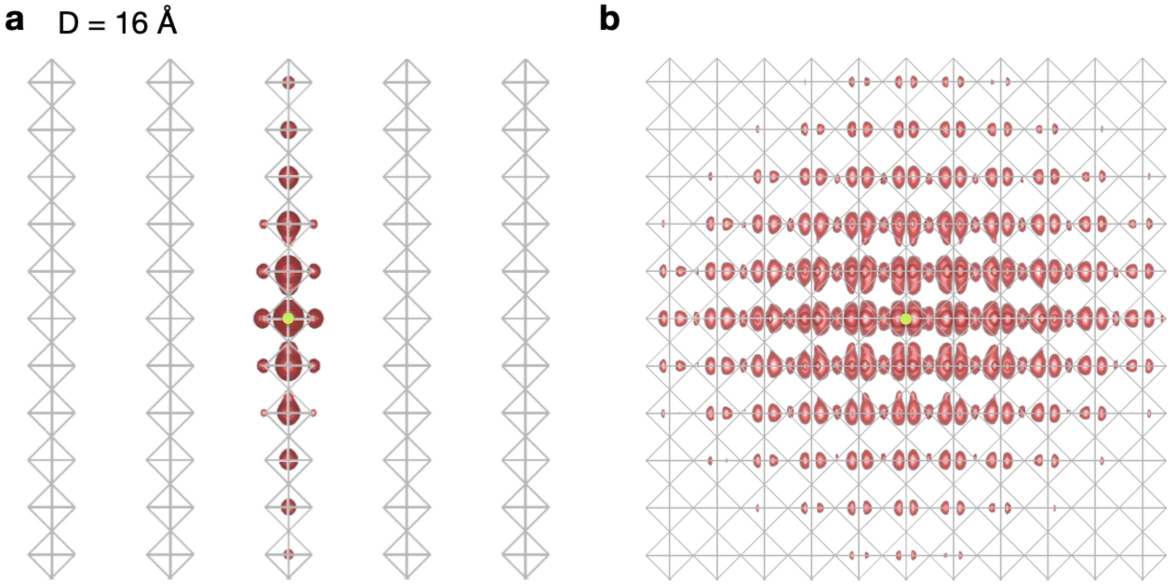

Figure S8: Isosurfaces representing the out-of-plane (a) and in-plane (b) spatial distribution of the lowest-lying exciton for a model DJ structure with an interlayer distance of 16 Å; the hole position is fixed at the center Pb atom of the central layer, marked by the green points.

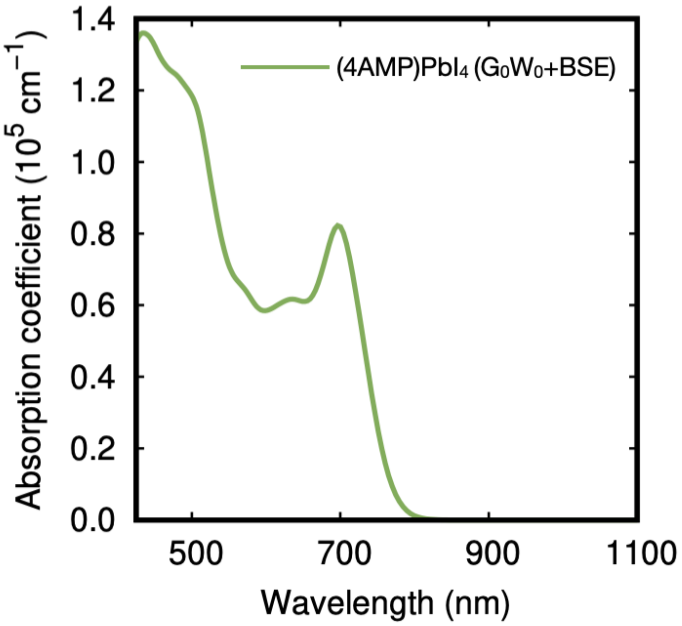

Figure S9: Optical absorption coefficient calculated for (4AMP)PbI4 within the G_0W_0+BSE framework. The spectrum is calculated by averaging over polarization directions. The onset of the spectrum is red-shifted with respect to experiment[18] by approximately 0.6 eV, consistent with prior studies[1] and as discussed in Section S2 of the SI.

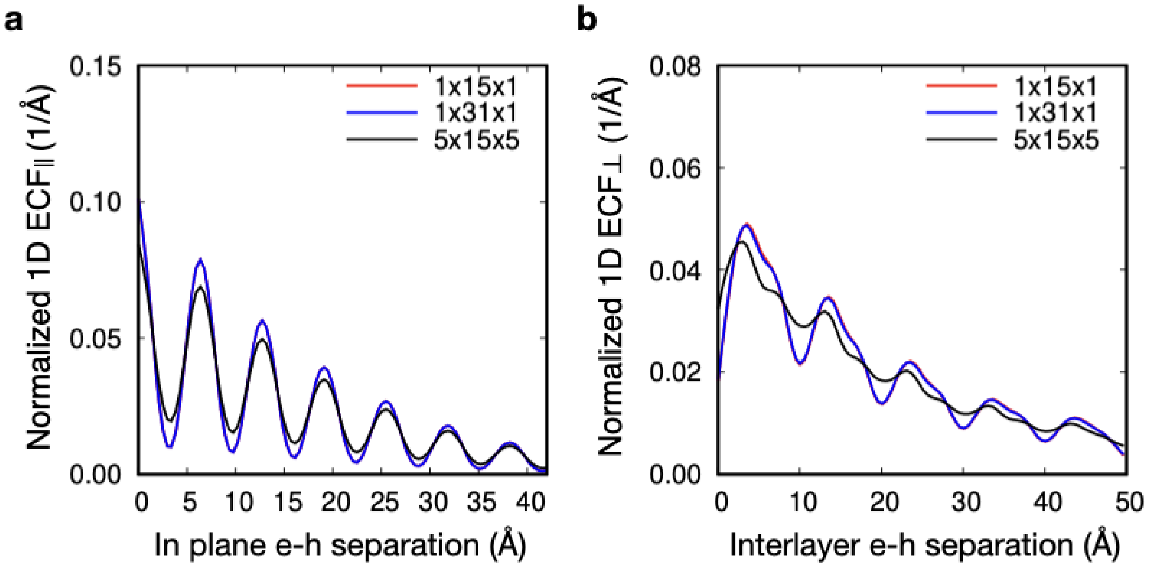

Figure S10: Normalized 1D ECFs for a model DJ structure with an interlayer distance of 10 Å, along the in-plane (a) and out-of-plane (b) direction, calculated with different hole sampling grids.

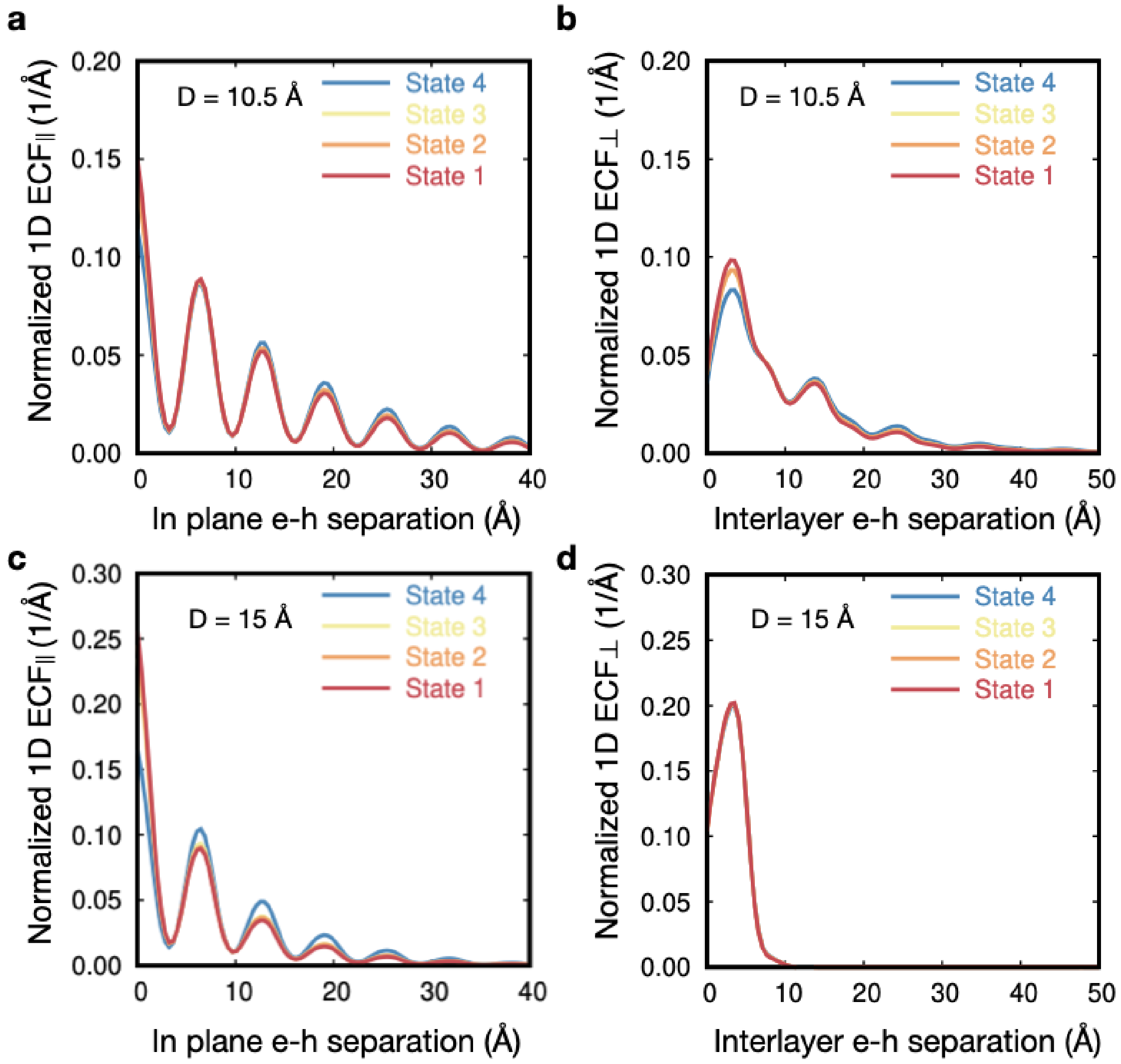

Figure S11: Comparison of the normalized 1D ECF for different excited states of 2 representative DJ model structures. (a) shows the ECF along the in-plane direction for D = 10.5 Å, while (b) represents the ECF along the out-of-plane direction. (c) and (d) show the ECF along in-plane and out-of-plane direction, respectively, for D = 15 Å.

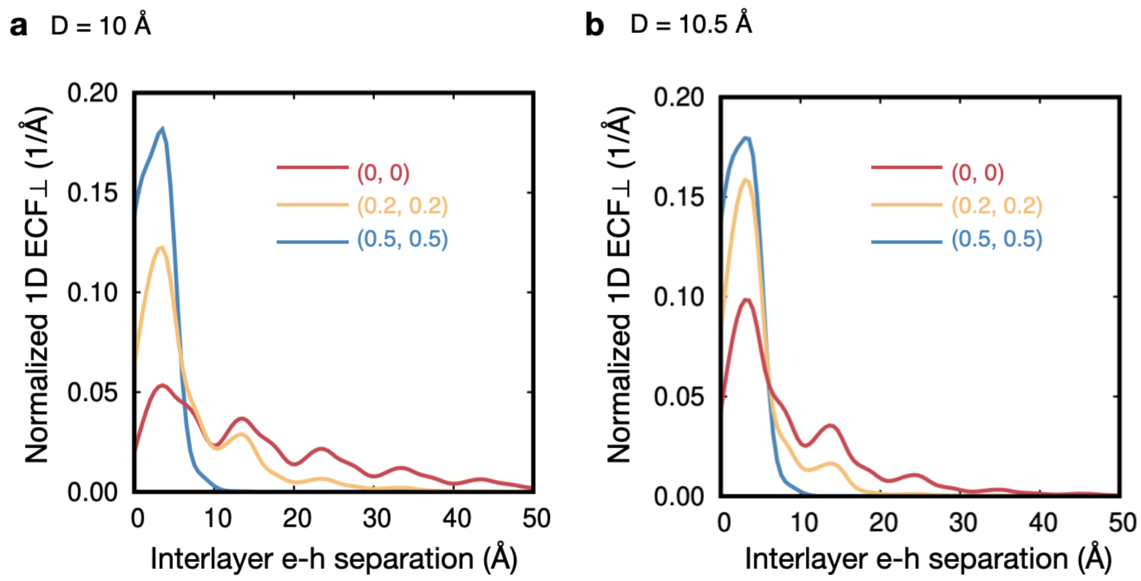

Figure S12: Normalized 1D ECF along the out-of-plane direction, for DJ model structures with different layer alignment and interlayer distances of 10 Å (a) and 10.5 Å (b).

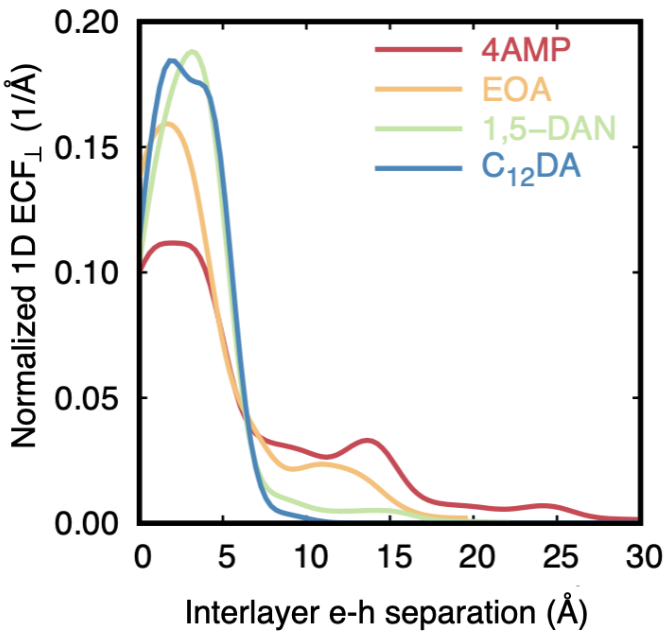

Figure S13: Normalized 1D ECF along the out-of-plane direction for 4 representative experimental structures as described in the main text.

References

(1) Filip, M. R.; Qiu, D. Y.; Del Ben, M.; Neaton, J. B. Screening of Excitons by Organic Cations in Quasi-Two-Dimensional Organic–Inorganic Lead-Halide Perovskites. *Nano Lett.* **2022**, *22*, 4870–4878.

(2) Hohenberg, P.; Kohn, W. Inhomogeneous Electron Gas. *Phys. Rev.* **1964**, *136*, B864–B871.

(3) Garrity, K. F.; Bennett, J. W.; Rabe, K. M.; Vanderbilt, D. Pseudopotentials for High-Throughput DFT Calculations. *Comput. Mater. Sci.* **2014**, *81*, 446–452.

(4) Giannozzi, P.; Andreussi, O.; Brumme, T.; Bunau, O.; Nardelli, M. B.; Calandra, M.; Car, R.; Cavazzoni, C.; Ceresoli, D.; Cococcioni, M., et al. Advanced Capabilities for Materials Modelling with Quantum ESPRESSO. *J. Phys. Condens. Matter* **2017**, *29*, 465901.

(5) Perdew, J. P.; Burke, K.; Ernzerhof, M. Generalized Gradient Approximation Made Simple. *Phys. Rev. Lett.* **1996**, *77*, 3865–3868.

(6) Hamann, D. R. Optimized Norm-Conserving Vanderbilt Pseudopotentials. *Phys. Rev. B* **2013**, *88*, 85117.

(7) Deslippe, J.; Samsonidze, G.; Strubbe, D. A.; Jain, M.; Cohen, M. L.; Louie, S. G. BerkeleyGW: A Massively Parallel Computer Package for the Calculation of the Quasiparticle and Optical Properties of Materials and Nanostructures. *Comput. Phys. Commun.* **2012**, *183*, 1269–1289.

(8) Godby, R. W.; Needs, R. J. Metal-Insulator Transition in Kohn-Sham Theory and Quasiparticle Theory. *Phys. Rev. Lett.* **1989**, *62*, 1169.

(9) Filip, M. R.; Haber, J. B.; Neaton, J. B. Phonon Screening of Excitons in Semiconductors: Halide Perovskites and Beyond. *Phys. Rev. Lett.* **2021**, *127*, 067401.

(10) Leppert, L.; Rangel, T.; Neaton, J. B. Towards Predictive Band Gaps for Halide Perovskites: Lessons from One-Shot and Eigenvalue Self-Consistent *GW*. *Phys. Rev. Mater.* **2019**, *3*, 103803.

(11) Gant, S. E.; Haber, J. B.; Filip, M. R.; Sagredo, F.; Wing, D.; Ohad, G.; Kronik, L.; Neaton, J. B. Optimally Tuned Starting Point for Single-Shot *GW* Calculations of Solids. *Phys. Rev. Mater.* **2022**, *6*, 053802.

(12) Rohlfing, M.; Louie, S. G. Electron-Hole Excitations in Semiconductors and Insulators. *Phys. Rev. Lett.* **1998**, *81*, 2312–2315.

(13) Rohlfing, M.; Louie, S. G. Electron-Hole Excitations and Optical Spectra from First Principles. *Phys. Rev. B* **2000**, *62*, 4927.

(14) Sharifzadeh, S.; Darancet, P.; Kronik, L.; Neaton, J. B. Low-Energy Charge-Transfer Excitons in Organic Solids from First-Principles: The Case of Pentacene. *J. Phys. Chem. Lett.* **2013**, *4*, 2197–2201.

(15) Mao, L.; Ke, W.; Pedesseau, L.; Wu, Y.; Katan, C.; Even, J.; Wasielewski, M. R.; Stoumpos, C. C.; Kanatzidis, M. G. Hybrid Dion–Jacobson 2D Lead Iodide Perovskites. *J. Am. Chem. Soc.* **2018**, *140*, 3775–3783.

(16) Lemmerer, A.; Billing, D. G. Lead Halide Inorganic–Organic Hybrids Incorporating Diammonium Cations. *CrystEngComm* **2012**, *14*, 1954–1966.

(17) Lemmerer, A.; Billing, D. G. Effect of Heteroatoms in the Inorganic–Organic Layered Perovskite-Type Hybrids [$(ZCnH_{2n}NH_3)_2PbI_4$], n = 2, 3, 4, 5, 6; Z = OH, Br and I; and [$(H_3NC_2H_4S_2C_2H_4NH_3)PbI_4$]. *CrystEngComm* **2010**, *12*, 1290–1301.

(18) Yin, J.; Naphade, R.; Maity, P.; Gutiérrez-Arzaluz, L.; Almalawi, D.; Roqan, I. S.; Brédas, J.-L.; Bakr, O. M.; Mohammed, O. F. Manipulation of Hot Carrier Cooling

Dynamics in Two-Dimensional Dion–Jacobson Hybrid Perovskites via Rashba Band Splitting. *Nat. Commun.* **2021**, *12*, 3995.
